# Vitamin D deficiency exacerbates UV/endorphin and opioid addiction

**DOI:** 10.1126/sciadv.abe4577

**Published:** 2021-06-11

**Authors:** Lajos V. Kemény, Kathleen C. Robinson, Andrea L. Hermann, Deena M. Walker, Susan Regan, Yik Weng Yew, Yi Chun Lai, Nicholas Theodosakis, Phillip D. Rivera, Weihua Ding, Liuyue Yang, Tobias Beyer, Yong-Hwee E. Loh, Jennifer A. Lo, Anita A. J. van der Sande, William Sarnie, David Kotler, Jennifer J. Hsiao, Mack Y. Su, Shinichiro Kato, Joseph Kotler, Staci D. Bilbo, Vanita Chopra, Matthew P. Salomon, Shiqian Shen, Dave S. B. Hoon, Maryam M. Asgari, Sarah E. Wakeman, Eric J. Nestler, David E. Fisher

**Affiliations:** 1 Cutaneous Biology Research Center, Department of Dermatology and Cancer Center, Massachusetts General Hospital, Harvard Medical School, Boston, MA, USA.; 2 Nash Family Department of Neuroscience and Friedman Brain Institute, Icahn School of Medicine at Mount Sinai, New York, NY, USA.; 3 Department of Medicine, Massachusetts General Hospital, Harvard Medical School, Boston, MA, USA.; 4 National Skin Centre, Singapore, Singapore.; 5 Rutgers New Jersey Medical School, Newark, NJ, USA.; 6 Program in Neuroscience, Harvard Medical School, Boston, MA, USA.; 7 Department of Pediatrics, Lurie Center for Autism, Massachusetts General Hospital for Children, Boston, MA, USA.; 8 Department of Biology, Hope College, Holland, MI, USA.; 9 MGH Center for Translational Pain Research, Department of Anesthesia, Critical Care and Pain Medicine, Massachusetts General Hospital, Harvard Medical School, Boston, MA, USA.; 10 USC Libraries Bioinformatics Services, University of Southern California, Los Angeles, CA, USA.; 11 Department of Neurology, Harvard Medical School, Massachusetts General Hospital, Charlestown, MA, USA.; 12 Department of Translational Molecular Medicine, Division of Molecular Oncology, John Wayne Cancer Institute at Providence Saint John’s Health Center, Santa Monica, CA, USA.; 13 Department of Dermatology, Massachusetts General Hospital and Department of Population Medicine, Harvard Medical School, Boston, MA, USA.

## Abstract

The current opioid epidemic warrants a better understanding of genetic and environmental factors that contribute to opioid addiction. Here we report an increased prevalence of vitamin D (VitD) deficiency in patients diagnosed with opioid use disorder and an inverse and dose-dependent association of VitD levels with self-reported opioid use. We used multiple pharmacologic approaches and genetic mouse models and found that deficiencies in VitD signaling amplify exogenous opioid responses that are normalized upon restoration of VitD signaling. Similarly, physiologic endogenous opioid analgesia and reward responses triggered by ultraviolet (UV) radiation are repressed by VitD signaling, suggesting that a feedback loop exists whereby VitD deficiency produces increased UV/endorphin-seeking behavior until VitD levels are restored by cutaneous VitD synthesis. This feedback may carry the evolutionary advantage of maximizing VitD synthesis. However, unlike UV exposure, exogenous opioid use is not followed by VitD synthesis (and its opioid suppressive effects), contributing to maladaptive addictive behavior.

## Teaser

Addiction to UV light and opioids is increased by VitD deficiency, suggesting that a feedback loop promotes its synthesis.

## INTRODUCTION

Opioid use disorder (OUD) is a major medical challenge that is continuing to increase in the United States. On the basis of the National Survey on Drug Use and Health, in 2018, approximately 10.3 million people aged 12 or older had misused opioids in the past year, and 2 million had an OUD (*1*). Abatement of the crisis will require more than a singular focus on opioid prescriptions and must include rapid expansion of effective treatments, including pharmacologic therapies for OUD, harm reduction interventions, and alleviation of social and economic determinants such as physical and psychological trauma and diminishing employment opportunities and life satisfaction (*2*). Therefore, causative but preventable environmental factors that contribute to opioid addiction are of great interest (*3*).

Human studies have suggested that ultraviolet (UV) tanning may be addictive (*4*, *5*), exhibiting characteristics highly reminiscent of opioid addiction (*6*). Recent preclinical data have identified an endogenous opioid-mediated addiction-like pathway triggered by UV-induced cutaneous synthesis of β-endorphin (*7*). Maintenance of UV-dependent vitamin D (VitD) synthesis has been suggested as a driver for the evolution of light skin pigmentation (*8*). Therefore, we hypothesized that UV-seeking behavior might be driven by VitD deficiency to maximize VitD synthesis and that VitD deficiency might also sensitize individuals to exogenous (UV-independent) opioids, contributing to opioid addiction. Moreover, a negative feedback loop might exist whereby UV/opioid-seeking behaviors are repressed when VitD levels are restored. This feedback might carry the evolutionary advantage of maximizing VitD synthesis. However, unlike UV exposure, exogenous opioid use is not followed by VitD synthesis (and its opioid suppressive effects), contributing to a maladaptive addictive behavior cycle.

## RESULTS

### VitD deficiency is associated with opioid use and OUD

Previous studies have shown associations of VitD deficiency with higher opioid doses among patients with opioid-consuming chronic pain (*9*) and patients with palliative cancer (*10*). These studies are confounded by the presence of pain, which is also associated with VitD deficiency (*11*). Therefore, we asked whether a relationship may exist between opioid use and serum VitD levels in humans, independently of pain, using data from the National Health and Nutrition Examination Survey [NHANES, 2003–2004, (*12*)]. As summarized in Table 1, compared to those with normal/sufficient serum VitD (>20 ng/ml), subjects with deficient (<12 ng/ml) or insufficient (12 to 20 ng/ml) VitD levels were more likely to use opioid painkillers, with unadjusted odds ratios (ORs) of 1.62 [95% confidence interval (CI) 1.07 to 2.45] and 1.27 (95% CI 0.91 to 1.78), respectively (*P* value for linear trend of 0.053), a notable pattern, but one likely complicated by several confounding factors, including age, sex, history of fractures, season of blood draw, and presence of chronic pain. Adjusting for those factors, the ORs for opioid painkiller use were 1.9 (95% CI 1.17 to 3.07) for deficient and 1.52 (95% CI 1.06 to 2.19) for VitD insufficient responders, *P* value for linear trend of 0.014. These data suggest an inverse, dose-dependent relationship between VitD signaling and opioid use, independent of known opioid use triggers.

**Table 1:** VitD deficiency is dose-dependently associated with opioid painkiller use. Analysis of the relationship between opioid analgesic consumption and VitD levels from the NHANES database was done using χ^2^ or Fisher’s exact tests. Multivariate analysis with logistic regression modeling was performed with opioid analgesic consumption as the dependent variable and with age, gender, ethnic groups, fracture history, chronic pain, season of blood draw, and VitD levels as the independent variables.

**Characteristics of NHANES cohort 2003–2004 with measured serum VitD levels by opioid exposure**								
**Characteristic**	**No opioid** **pain killer use**	**Opioid** **painkiller use**	**ORs**	**95% CI**	***P* value**	**Adjusted OR**	**95% CI**	***P* value**
**Age** (at blood draw) means ± SD (range)	43.5 ± 28.0	51.8 ± 21.7	1.01	1.01–1.02	<0.001	0.99	0.97–1.00	0.025
**Gender**				0.77–1.13	0.475			0.783
Female	9503	227	1.00			1.00		
Male	8407	187	0.93			1.04	0.77–1.42	
**Race**					<0.001			0.325
Non-Hispanic White	9123	252	1.00			1.00		
Hispanic	4142	66	0.58	0.44–0.76		0.69	0.43–1.12	
Non-Hispanic Black	3927	85	0.78	0.61–1.01		0.76	0.49–1.18	
Other*	718	11	0.56	0.30–1.02		0.68	0.31–1.50	
**History of** **fracture**					<0.001			0.075
Yes	1829	88	1.74	1.36–2.22		1.42	0.97–2.09	
No	10339	286	1.00			1.00		
**Serum VitD** **levels—** **categorical**					0.053			0.014
Deficient (<12 ng/ml)	615	32	1.62	1.07–2.45		1.90	1.17–3.07	
Insufficient (12–20 ng/ml)	1395	57	1.27	0.91–1.78		1.52	1.06–2.19	
Normal (≥20 ng/ml)	2866	92	1.00			1.00		
**Season of** **blood draw**					0.960			0.929
Nov 1 to Apr 30	8007	183	1.00			1.00		
May 1 to Oct 31	8923	205	1.01	0.82–1.23		0.99	0.71–1.36	
**Chronic pain** **(>1 year)**					<0.001			<0.001
Yes	2308	159	3.16	2.56–3.90		2.66		
No	9854	215	1.00			1.00	1.91–3.69	

*Asians, Native Americans, and Pacific Islanders, and multiracial categories.

As continuous high opioid intake could increase the risk of OUD (*13*), we hypothesized that VitD deficiency might be associated with this condition. Among primary care patients at Massachusetts General Hospital in calendar years 2014–2016 (*N* = 163,531), we compared patients with OUD (cases, *N* = 2772) to control patients without OUD diagnosis matched on age (within 5 years), sex, race, and primary care provider, on a 1:3 basis (*N* = 8265). The results are presented in Table 2.

**Table 2:** VitD deficiency is associated with OUD. Analysis of VitD levels in a patient population seen at primary care practices at Massachusetts General Hospital (MGH) in calendar years 2014–2016 (*N* = 163,531). Patients were classified as cases if they received a diagnosis code for OUD during this period. Cases were matched to control patients without diagnosis codes on age (within 5 years), sex, race, and primary care provider on a 3:1 basis. Distribution of VitD levels between cases and controls was compared using χ^2^ tests. Linear regression was used to assess the effect of case status on VitD measurement controlling for demographics.

		**Case (*n* = 2772)**	**Case (%)**	**Control (*n* = 8265)**	**Control (%)**	***P* value**
**Any measurement** **available**		1169	42%	3491	42%	0.951
**Number of** **measurements**	1	459	39%	1270	36%	
	2	237	20%	687	20%	
	3	145	12%	432	12%	0.342
	4	90	8%	299	9%	
	≥5	238	20%	803	23%	
**Age (≥50 years)**		713	61%	2132	61%	0.962
**Race/ethnicity**	White	993	85%	3042	87%	0.199
	Black	62	5%	150	4%	
	Hispanic	46	4%	135	4%	
	Other	68	6%	164	5%	
**Sex (female)**		597	51%	1872	54%	0.13
**Most recent** **measurement**	Mean (SD)	29.1 (12.8)		32.2 (13.2)		<0.001
	<12	42	4%	73	2%	
	12–20	283	24%	531	15%	
	21–30	362	31%	1097	31%	
	>30	482	41%	1790	51%	
**Mean of all** **measurements**	Mean (SD)	29.01 (11.6)		30.8 (11.0)		<0.001
**Ever deficient** **status (≤20)**		493	42%	1285	37%	0.001

The distribution of VitD levels differed by case status (*P* < 0.001), with more cases than controls classified as insufficient or deficient (28% versus 17%) and fewer classified as optimal for VitD (41% versus 51%). Cases were more likely than controls to have ever had a deficient VitD level (42% versus 37%, *P* = 0.001).

### VitD deficiency increases sensitivity to morphine reward

**Fig. 1:**
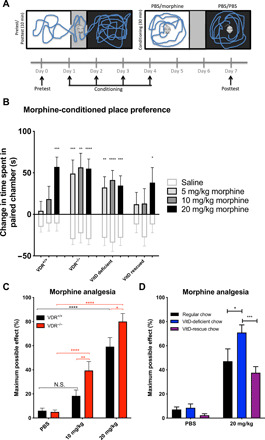
Deficiencies in VitD signaling increase morphine-induced place preference and analgesia. (**A**) Mice (10 to 12 per group) were conditioned to different doses of morphine or saline in the white chamber and to saline in the black chamber, and place preferences were evaluated as change in time spent in the white chamber postconditioning versus preconditioning. (**B**) Different doses of morphine were compared individually with their corresponding saline groups. Note that certain morphine-treated mice have identical saline groups, as those experiments were conducted simultaneously. Data are represented as the means ± SEM, *P* values were obtained by two-way analysis of variance (ANOVA) with Sidak’s multiple comparisons test. *P < 0.05 compared with the corresponding saline group. (**C**) Mice intraperitoneally received a single dose of PBS (*n* = 16 per group), morphine (10 mg/kg) (*n* = 12 and 11 for *Vdr*^−/−^ and *Vdr*^+/+^, respectively), or morphine (20 mg/kg) (*n* = 15 and 20 for VDR^−/−^ and *Vdr*^+/+^, respectively). N.S., not significant. (**D**) Similarly to (C), mice received PBS or morphine (20 mg/kg), and then analgesia was measured using thermal nociception assay. *n* = 10, 9, and 10 for regular, VitD-deficient, and VitD-rescued groups, respectively. Analgesia was calculated as maximum potential effect described in Materials and Methods in details for (C) and (D). Data are represented as the means ± SEM, *P* values were obtained by two-way ANOVA with Sidak’s multiple comparisons test. **P < 0.01, ***P < 0.001, and ****P < 0.0001.

To investigate whether VitD deficiency can be a causative (and preventable) factor in these associations and lead to greater opioid consumption through sensitizing to opioid addiction, first, we tested the reinforcing effects of morphine by conditioned place preference (CPP) in multiple mouse models (Fig. 1A). To establish VitD deficiency, mice were fed a VitD-devoid diet for at least 8 weeks [serum 25(OH) Vit D3 levels are shown in fig. S1]. Some VitD-deficient mice were “rescued” by returning them to the normal VitD-containing diet for at least 8 additional weeks. In addition, we used VitD receptor (VDR) knockout mice to genetically model deficient VitD signaling. Morphine produced dose-dependent increases in CPP responses in control mice with normal VitD levels (Fig. 1B), with strong morphine-induced CPP observed only at the highest dose of 20 mg/kg. In contrast, both *Vdr*^−/−^ and VitD-deficient mice exhibited strong morphine preferences at all doses tested, with an at least fourfold left shift of the dose-response curve compared with wild-type mice suggesting increased preference. Oral supplementation to rescue VitD deficiency restored the morphine preference pattern back to that of VitD-replete mice (Fig. 1B), demonstrating that the VitD-dependent effect is reversible.

We repeated the morphine CPP experiment in an alternative CPP setup that has tactile cues in addition to the visual cues, making it easier to associate the rewarding cues with the rewarding stimuli (see Materials and Methods for details). In these experiments, we used a very low dose of morphine (0.25 mg/kg) that does not change place preference in VitD receptor wild-type and VitD-rescued animals (fig. S2). However, both *Vdr*^−/−^ and VitD-deficient mice spent more time in the morphine-paired chamber compared with the saline-paired chamber after low-dose morphine conditioning.

We attempted to do a morphine CPP assay using VDR wild-type mice with “normal” VitD levels that had received calcitriol treatment to investigate whether the development of CPP could be prevented if VDR signaling was enhanced by calcitriol injections. However, we did not see a decrease of morphine CPP in calcitriol treated animals (fig. S3). This could be due to the already “high” VitD levels present in these mice or another possibility is that the effects of VitD supplementation might require a longer time to impact opioid responses. These results collectively support the hypothesis that deficiencies in VitD receptor signaling sensitize animals to opioid-seeking behavior that is reversible upon VitD supplementation.

### VitD signaling regulates nociception and opioid analgesia

To examine whether VitD deficiency might alter additional opioid-induced behaviors, we first measured basal thermal nociceptive thresholds. We observed that diminished VitD signaling, produced by either VitD deficiency or VitD receptor knockout, produced significantly increased basal thermal nociceptive thresholds, which were rescued in VitD-deficient mice by oral VitD replenishment (fig. S4A). We observed that the elevated nociceptive threshold required the presence of mu opioid receptors (OPRM1), because it was abrogated in OPRM1-null mice (fig. S4B). Peripheral administration of the opioid receptor antagonist naltrexone reversed the elevated thresholds observed in *Vdr*^−/−^ mice (fig. S4C). However, peripheral injection of methylnaltrexone, a methylated form of naltrexone that does not cross the blood-brain barrier and thus blocks opioid receptors only peripherally (*14*), did not reverse the elevated nociceptive thresholds of *Vdr*^−/−^ mice (fig. S4D). Together, these results suggest that the absence of VitD signaling increases nociceptive thresholds via central opioid signaling.

It has been observed previously that analgesic sensitivity to opioids can be predicted from higher initial nociceptive and pain thresholds in mice and humans (*15*, *16*). We therefore investigated whether VitD deficiency would also result in higher opioid analgesia and found that *Vdr^−/−^* mice showed greater morphine analgesia than wild-type mice, even at a morphine dose of 10 mg/kg, which did not produce significant analgesia relative to phosphate-buffered saline (PBS)/vehicle control in wild-type mice (Fig. 1C). Similarly, dietary VitD deficiency sensitized to the acute analgesic effects of morphine, whereas VitD rescue reversed this effect (Fig. 1D and fig. S5).

Repeated exposure to opioids results in the development of tolerance, characterized by progressive loss of an analgesic effect. This may lead to the need for higher opioid doses to achieve similar levels of analgesia and increased total opioid consumption, simultaneously limiting clinical efficacy of opioids and increasing the likelihood of dependence and addiction. We investigated the effect of VitD signaling on the kinetics of tolerance induction, using a thermal nociception assay. With daily morphine injections, mice lacking VitD receptor showed significantly decreased analgesia when injected the second time with morphine, whereas wild-type mice did not show a reduction in analgesia until the fourth day (fig. S6, A, C, and E). Dietary VitD deficiency had similar, but milder, effects as VDR knockout compared with wild-type mice (fig. S6, B, D, and F). Collectively, these observations corroborate observations made more than 30 years ago of increased basal pain thresholds, analgesic effects, and faster tolerance to morphine in VitD-deficient rats (*17*).

### VitD deficiency exacerbates opioid dependence

**Fig. 2:**
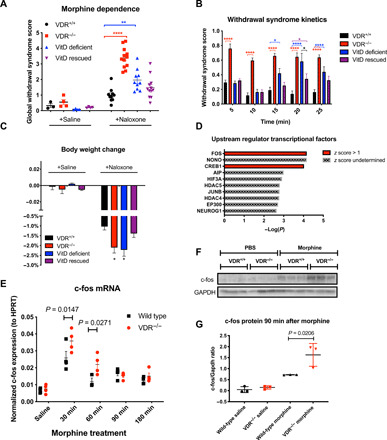
Morphine dependence and morphine-induced accumbal c-fos expression is greater in the absence of VitD signaling. To investigate morphine dependence, acute morphine withdrawal was precipitated by intraperitoneal injection of naloxone in morphine dependent mice (see Materials and Methods for details). Immediately after injection of naloxone, mice were placed in a plexiglass cylinder, and their behavior was monitored for 25 min for the appearance of nine withdrawal symptoms, from which a composite global withdrawal score (**A**) was calculated. Three to four mice from each group served as nonwithdrawn controls that received saline injections instead of naloxone. Individual withdrawal signs and the kinetics of withdrawal revealed differences between VitD-deficient models and wild-type controls [(**B**) and figs. S7 and S8]. Weight loss after naloxone-precipitated withdrawal was greater in the VitD-deficient models (**C**), *n* = 3, 4, 4, 4, 10, 12, 7, and 12 per group in the order of display. Data are represented as the means ± SEM. *P < 0.05 by one-way ANOVA with Dunnett’s multiple comparisons test (body weight change) or Kruskal-Wallis test with Dunn’s multiple comparisons test (all other measures) compared to naloxone-treated control groups. (**D**) Ingenuity Pathway Analysis of upstream transcriptional regulators of morphine-induced differentially expressed genes between *Vdr*^−/−^ and wild-type mice in the nucleus accumbens. Top 10 predicted regulators are ranked by predicted activation score (*z* score) and −log(*P*) values. (**E**) Morphine induced significantly higher c-Fos mRNA induction in the nucleus accumbens in *Vdr*^−/−^ mice compared with wild-type mice. HPRT, hypoxanthine-guanine phosphoribosyltransferase. (**F** and **G**) Morphine-induced c-Fos protein expression is significantly higher in *Vdr*^−/−^ mice compared with wild-type mice. Data are represented as the means ± SEM; *P* values were obtained by two-way ANOVA with Sidak’s multiple comparisons test. *P < 0.05 compared with the corresponding saline group. GAPDH, glyceraldehyde phosphate dehydrogenase. **P < 0.01 and ****P < 0.0001.

Another component of addiction is the development of drug dependence, which is characterized by physical withdrawal symptoms upon abrupt drug abstinence or receptor antagonism. After inducing opioid dependence by administering escalating doses of morphine over 7 days, opioid withdrawal was precipitated by naloxone (1 mg/kg). Multiple opioid withdrawal behaviors were monitored and quantified for 25 min after naloxone administration, and a composite global withdrawal score was calculated (see Materials and Methods for details). Additional mice received saline injections instead of naloxone that showed negligible dependence than mice where withdrawal was precipitated with naloxone (Fig. 2A). Morphine dependence was significantly greater in *Vdr*^−/−^ and VitD-deficient mice compared with wild-type mice (Fig. 2A). Rescuing VitD partially reversed the increased dependence in VitD-deficient mice. We also observed that the kinetics of withdrawal symptoms showed a distinct pattern with VitD deficiency, wherein some of the symptoms occurred after a delay and peaked ~20 min after naloxone injection (Fig. 2B, blue bars). The rescued mice showed even greater delay of withdrawal symptoms; however, VitD partially reversed the increased dependence. Individual withdrawal symptoms revealed similar patterns among the different groups (figs. S7 and S8). In addition, significantly greater acute weight loss, a surrogate for fluid loss through diarrhea, was observed in *Vdr*^−/−^ and VitD-deficient mice compared with wild-type or VitD-rescued mice (Fig. 2C). These results suggest that deficiencies in VitD signaling are associated with greater physical dependence to opioids, which can be alleviated by correcting VitD levels.

### Opioid-induced c-fos transcription in the nucleus accumbens is repressed by VDR

To examine the mechanism underlying the increased morphine reward in *Vdr*^−/−^ mice, we performed RNA sequencing (RNA-seq) on bilateral micropunches of the nucleus accumbens, a key brain region for morphine reward, from *Vdr*^−/−^ and wild-type mice 90 min after intraperitoneal injection of PBS or morphine sulfate (5 mg/kg). As expected, morphine-induced gene expression changes in multiple pathways involved in opioid signaling, including targets of cyclic adenosine 3′,5′-monophosphate (cAMP), Gα_i_, and cAMP response element–binding protein (CREB) signaling, in both wild-type and *Vdr*^−/−^ mice compared with PBS treatment (files S1 and S2). Next, to mechanistically examine increased morphine sensitivity in *Vdr*^−/−^ mice, we investigated the differentially expressed genes between morphine-treated *Vdr*^−/−^ and wild-type mice. Using upstream regulator analysis (see Materials and Methods for details), a tool that infers transcription factor activity based on expression patterns of target genes, we found that only FOS and CREB1 were predicted to be activated by morphine (*z* score > 1) in *Vdr*^−/−^ mice compared to wild-type mice (Fig. 2D and file S3). In morphine naïve (saline-treated) *Vdr*^−/−^ mice, FOS activity was not induced (*z* = −0.518) (file S4), suggesting that the increased FOS activation in *Vdr*^−/−^ mice is a result of morphine treatment.

Having observed greater c-FOS transcription factor target gene expression in the nucleus accumbens of *Vdr*^−/−^ animals (Fig. 2D and file S3), we investigated the kinetics of morphine-induced c-Fos mRNA and protein levels in the nucleus accumbens, where c-Fos has been shown to be required to elicit opioid reward (*18*). We found that maximal morphine induction of *c-Fos* mRNA was significantly higher in *Vdr*^−/−^ mice compared with wild-type animals 30 and 60 min following injection (Fig. 2E). The greater *c-Fos* mRNA induction was accompanied by higher induction of c-FOS protein expression in *Vdr*^−/−^ mice 90 min after morphine administration, the time point at which RNA-seq demonstrated increased c-FOS transcription factor activity (Fig. 2, F and G). The increased expression of *c-Fos* mRNA and protein suggest increased activity of the nucleus accumbens in response to morphine. In line with this, we observed that morphine induces increased brain-derived neurotrophic factor (BDNF) mRNA synthesis in the NAcc in *VDR^−/−^* mice in response to morphine (fig. S9). Because of relatively low expression of VDR in our RNA-seq data, coupled with its very low/absent expression in the nucleus accumbens in human brain samples (fig. S10), these data suggest that VDR regulates morphine-induced nucleus accumbens activity via a noncell-autonomous pathway, such as increased activation of input to the nucleus accumbens.

We have focused on multiple brain regions (for details, see Materials and Methods) and found that in *VDR^−/−^* mice, morphine-induced greater c-fos expression changes in the ventral tegmental area (VTA) compared with *Vdr* wild-type mice (fig. S11, A and B). Given previous findings that VitD signaling influences dopamine signaling in the VTA in mice (*19*), we performed RNA-seq of the VTA from VDR^−/−^ and wild-type mice after saline or morphine injections (files S5 to S8). In line with prior observations (*19*), we have observed a down-regulation of genes involved in dopamine signaling in the absence of VitD signaling (fig. S11, C and D) in saline-treated animals. This finding is in agreement with a recent report that measured decreased dopamine levels in VitD-deficient mice in the VTA and also with other studies that implicated the role of VitD in regulating dopamine metabolism (*20*–*23*). Therefore, it is possible that there are compensatory mechanisms downstream of dopamine receptor signaling that contribute to increased opioid reward in the VitD-deficient setting. Alternatively, nondopaminergic mechanisms [i.e., glutamatergic transmission from the Basolateral amygdala (BLA)] might contribute to the phenotype.

Next, we investigated the mRNA expression of OPRM1 in the VTA. Using the Allen Brian Atlas, we found a significant inverse correlation between VDR and OPRM1 mRNA in the VTA (fig. S11E), which is in line with our results of having increased opioid signaling in the absence of VDR signaling. However, we could not find significantly higher OPRM1 expression in the VTA in *Vdr*^−/−^ mice (fig. S11F), suggesting that either the correlation does not exist in mice or that some other brain areas might have altered OPRM1 expression in *Vdr*^−/−^ mice.

Although c-fos staining intensity was found to be greater in *Vdr*^−/−^ mice, upstream regulator analysis of the global transcriptome changes did not reveal as large of a c-fos activation in *Vdr*^−/−^ mice compared with wild-type mice in the VTA (*z* score = −0.4; file S7), as we have seen in the NAC (*z* score = +1.1; Fig. 2D and file S3). Collectively, these results suggest that in addition to the VTA, other brain regions likely also contribute to the increased activation of NAC. In addition, a recent study showed that VDR expression in the basolateral amygdala is negatively associated with the addiction index derived from multiple cocaine self-administration behaviors in female mice (*24*). This highlights the complexity of VitD signaling in reward behaviors and is in line with our observations that VitD deficiency likely modulates opioid responses in multiple brain areas.

Given the role of VitD signaling in morphine reward, we asked whether VDR might have a more general role in regulating reward responses to non-opioid drugs of abuse. We tested rewarding effects of nicotine, a non-opioid substance whose rewarding effects are modulated by opioid signaling (*25*). We observed strong and highly significant CPP for nicotine in *Vdr*^−/−^ mice compared with wild-type mice (fig. S12A). Prior observations showed that nicotine CPP is high in younger mice and decreases with age (*26*). In line with this pattern, the increased nicotine sensitivity in the setting of VDR knockout was age-dependent, as the same dose of nicotine did not elicit CPP in older (8-month) mice (fig. S12B).

### VitD deficiency increases UV radiation–induced endogenous analgesia and reward

**Fig. 3:**
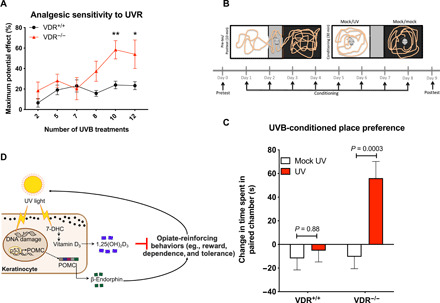
The absence of VDR signaling increases UV radiation–induced analgesia and UV CPP. (**A**) Daily UV radiation (UVR)–induced analgesia is significantly higher in *Vdr*^−/−^ mice compared with *Vdr*^+/+^ mice (*n* = 12 and 9, VDR^+/+^ and VDR^−/−^, respectively). (**B**) Mice were conditioned to UVB (*n* = 12 and 9, for VDR^+/+^ and VDR^−/−^, respectively) or mock UV treatment (*n* = 12 and 11, for VDR^+/+^ and VDR^−/−^, respectively) in the white chamber and to mock UV in the black chamber, and place preferences were evaluated as change in time spent in the white chamber postconditioning versus preconditioning. (**C**) VDR represses chronic low-dose UV radiation–induced place preference. Data are represented as the means ± SEM, *P* values were obtained by two-way ANOVA with Sidak’s multiple comparisons test. *P < 0.05 compared with the corresponding saline group. (**D**) Model depicting UV radiation–induced behavior changes that are inhibited by a negative feedback loop by VitD synthesis. POMC, proopiomelanocortin. 7-DHC, 7-Dehydrocholesterol. **P <0.01.

Last, we asked whether VitD deficiency might sensitize to physiologic endogenous opioid signaling in response to UV radiation. Because the addiction-like effects of UV were previously shown to be mediated by endorphin/opioid signaling (*7*), we anticipated that the VDR^−/−^ state would derepress opioid/CPP responses. We used *Vdr*^−/−^ mice to ablate effects of UV-induced de novo cutaneous VitD synthesis. Similar to increased exogenous opioid analgesia, daily low-dose UV (50 mJ/cm^2^) induced significantly higher analgesia in *Vdr*^−/−^ mice compared with wild-type mice (Fig. 3A). Next, we examined whether VitD deficiency might alter UV-seeking behavior. Mice were conditioned with either UV or mock radiation in a CPP apparatus and tested for place preference (Fig. 3B). UV exposure induced a strong CPP in the *Vdr*^−/−^ mice, in contrast to a minimal and insignificant trend toward preference in *Vdr* wild-type mice (Fig. 3C). UV has numerous effects that are independent of VitD; it is notable that the UV responses observed here in *Vdr*^−/−^ were phenocopied by morphine treatments in VitD deficient as well as *Vdr*^−/−^ mice, suggesting that the CPP effects were opiate/opioid mediated and thus not strictly UV dependent—and they were also reversible with restoration of VitD levels. These results suggest that the lack of VitD signaling sensitizes individuals to the rewarding effects of UV, in line with an adaptive feedback loop in which deficiencies in VitD signaling increase UV/opioid reward to maximize VitD synthesis, whereas correcting VitD signaling restores normal sensitivity to UV (illustrated in Fig. 3D).

## DISCUSSION

Evolutionarily, developing addiction to ultraviolet B (UVB) radiation, a ubiquitous carcinogen whose effects manifest mainly in postreproductive years, could increase fitness of a population if accompanied by positive effects in childhood or early adulthood. The detrimental health effects of VitD deficiency have been suggested to contribute as an evolutionary driver for light skin pigmentation in humans (*8*), and it is plausible that additional mechanisms, like UV-seeking behavior, may further contribute to maintenance of VitD levels in humans and other species. Panther chameleons optimize natural sunlight exposure by fine-tuning basking behavior, depending on their VitD status (*27*). VitD deficiency–associated modulation of endogenous opioid sensitivity and reward might have evolved to promote sun-seeking behavior that replenishes VitD levels that are essential during development and growth, despite late negative consequences of accelerated skin photoaging and skin cancer. However, consumption of exogenous opioids does not trigger VitD synthesis and consequent suppression of opioid sensitivity but may instead produce an amplifying cycle of dependence.

Mechanistically, increased c-fos activation in the nucleus accumbens in the absence of VitD signaling is in line with our observations that opioids induce greater CPP and opioid dependence in VitD-deficient backgrounds. While the exact mechanism that causes greater nucleus accumbens activation remains unclear, several possible mechanisms exist. VDR is ubiquitously expressed in multiple brain regions; therefore, it is plausible that VDR represses opioids simultaneously in multiple regions (i.e., VTA, BLA, etc.). Future functional studies modulating VDR signaling in these regions could clarify in which regions VDR signaling is necessary and sufficient to modulate opioid reward.

We have seen that dopamine signaling is altered in the VTA of *Vdr*^−/−^ mice, in line with previous observations (*19*). It is possible that there are compensatory mechanisms downstream of dopamine receptor signaling that contribute to increased opioid reward in the VitD–deficient (and dopamine deficient) setting. Alternatively, nondopaminergic mechanisms (i.e., glutamatergic transmission from the BLA) might contribute to the phenotype.

The roles of VDR and VitD in regulating the immune system and redox homeostasis are well established. Therefore, future studies are warranted to explore the role of VitD signaling in various immune cell populations, like microglial cells, to investigate their potential role in opioid addiction. The inverse dose-response relationship between VitD levels and likelihood of opioid use, coupled with our preclinical data, is consistent with a model in which even modest rescue of VitD deficiency could be beneficial in the prevention and treatment of opioid addiction, especially considering that VitD is generally inexpensive, accessible, and safe.

It is an important clinical question to determine which patient subpopulation(s) would benefit most from VitD supplementation. It is unclear whether the critical VitD level, above which no additional benefit could be observed, might differ between species. The range of normal VitD levels in humans is quite wide, and we believe that future studies should examine this question within a clinical context because there exists a possibility that patients with low-normal VitD levels might benefit from additional VitD supplementation.

Although effective medication treatments for OUD exist, in real world, evaluations treatment retention remains challenging, and further interventions to augment the efficacy of these existing therapies are needed. In addition, limited data exist on effective interventions to prevent the development of OUD. Our findings offer several different therapeutic opportunities: Our results imply that VitD-deficient individuals may be at risk for developing tolerance and physiologic opioid dependence more rapidly, experiencing more significant withdrawal, and experiencing greater reward from opioid exposure. VitD supplementation might have a preventative benefit by decreasing opioid reward and possibly diminishing the risk of OUD. VitD supplementation may also improve the beneficial effects of medications for OUD. The alarming prevalence and toll of untreated OUD warrants timely clinical studies to test these therapeutic approaches directly, especially given the safety and availability of VitD supplementation.

## MATERIALS AND METHODS

### Mice and diets

All mice used in this study were on a C57BL/6 background. For selected experiments, mice with homozygous deletion of the C terminus of the proopiomelanocortin (POMC) gene, resulting in lack of β-endorphin (β-endorphin^−/−^) (*28*), were used. *Vdr*^−/−^ mice were provided by M. Damay (*29*). *Oprm1*^−/−^ mice were obtained from the Jackson laboratory (*30*).

VitD deficiency was produced by withdrawing VitD from the diet (Research Diets Inc., New Brunswick, NJ) for at least 2 months, beginning at 3 weeks of age. *Vdr*^−/−^ mice were raised on a diet with extra calcium (20% lactose, 2% calcium, 1.25% phosphate, Harlan Bioproducts, 96348) to allow adequate skeletal calcification.

VitD-rescued mice were on the VitD-deficient diet for 2 months, and then VitD was added back to the diet for at least 2 months. All mice were habituated to the holding room for at least a week before starting experiments. On the day of the behavior experiments, mice were moved and habituated to the procedure rooms for at least 30 min before starting experiments.

Experiments and analyses were carried out blindly where it was possible. All animals were used in the study, except for animals that suffered injuries that precluded them from providing data points thus were not included in any measurements. All studies and procedures involving animal subjects were performed in accordance with policies and protocols approved by the Institutional Animal Care and Use Committee at Massachusetts General Hospital.

### UV irradiation

Mice were dorsally shaved 1 day before the start of radiation exposure. Mice were reshaved if there were patches of fur regrowth, once every 2 weeks. To assess UV-induced analgesia, baselines were acquired before UV exposure. Then, mice were exposed daily to 50 mJ^2^ of UVB (using G15T8E UVB bulbs with peak emission of 305 to 310 nm) in the afternoon hours, and analgesia was assayed as described below. For UV-CPP, mice were exposed to 50 mJ/cm^2^ per day of UVB (an empirically determined sub-erythematic dose) for 5 days, followed by 25 mJ/cm^2^ per day of UVB 5 days/week (Monday to Friday) for 2 weeks. UVB was then applied every day (Monday to Sunday) in incrementally increasing doses (+5 mJ/cm^2^ every 4 days) until reaching 50 mJ/cm^2^, followed by 50 mJ/cm^2^ every day for 1 week.

### Blood draws and measurement of serum VitD levels

Mice were placed in a standard restrainer, and tail vein blood was collected in EDTA microvette tubes containing 0.6 Trypsin Inhibitory Units (TIU) aprotinin. Blood was drawn in the morning hours between 10 a.m. and 12 noon. Tubes of collected blood were kept on ice until centrifugation at 3500 rpm at 4°C for 20 min. Plasma was isolated and then stored at −80°C until VitD measurements. The 25(OH)-vitamin D3 was quantified by radioimmunoassay (Immuno-Biological Laboratories Inc., MN, USA).

### Nociceptive and analgesia assays

The hot plate test (*31*) was used to study thermal nociception and analgesia. Nociception tests were carried out as before (*7*). Briefly, mice were placed on a 52°C hot plate, and time of response (jumping, paw flutter, or paw licking) was measured. On three consecutive days before experiments, mice were habituated to the hot plate and the wire mesh rack 2 mi/day. For certain experiments, mice were injected with 10mg/kg dose of methylnaltrexone or naltrexone 60 min before a hot plate test was performed.

### Measurement of analgesia induced by UV and morphine

After establishing baseline thermal nociceptive thresholds, mice were injected with morphine (10 or 20 mg/kg) or with PBS vehicle as control. Nociceptive thresholds were measured 30 min after injections. Analgesia was calculated as percent maximum possible effect (MPE): MPE = (test latency − baseline latency)/(cutoff latency − baseline latency) (*32*), using 30s as a cutoff latency to prevent tissue damage. UV analgesia was measured similarly to morphine analgesia. Mice were exposed to UV irradiation (50 mJ/cm^2^) on a daily basis in the evening hours, and thermal nociceptive thresholds were measured in the following morning using the hot plate assay. Analgesia was calculated as for morphine.

### Measurement of morphine tolerance

Development of tolerance was established by daily intraperitoneal injections of morphine (20 mg/kg) (*33*). Analgesia was measured daily, 30 min after morphine injections, and was normalized to MPE measured on the first day of injection (referred to as day 0).

### Measuring morphine withdrawal

Escalating doses of morphine were administered intraperitoneally for six consecutive days (10, 20, 40, 60, 80, and 100 mg/kg twice daily), and then physical withdrawal symptoms were precipitated by intraperitoneal injection of naloxone (1 mg/kg) on the seventh day, 120 min after a single dose of morphine (100 mg/kg). After the injection of naloxone, mice were immediately placed in a 30 cm by 15 cm by 15 cm plexiglass cylinder, and their behavior was monitored for 25 min. Opioid withdrawal symptoms were evaluated blindly, where it was possible. Wet dog shakes, paw tremors, paw licks, bouts of grooming, jumping, chewing, and rearing events were counted. Wet eye and abnormal posture were quantified as occurrences within 5-min intervals, shaking within 15-s intervals. Diarrhea was estimated by counting the fecal boli. Weight loss was measured by weighing the mice before and 1 hour after naloxone injection. Global withdrawal syndrome and kinetics of withdrawal symptoms were calculated as a composite of the withdrawal symptoms (*34*); for each symptom, the mean value of the VitD control group was defined as 1, and the corresponding value for each mouse was then normalized to this mean to obtain a score for each withdrawal symptom. Next, for each mouse, the scores of these nine withdrawal symptoms were then averaged to obtain the global withdrawal syndrome score. To obtain the kinetics of withdrawal symptoms, for each individual mouse, the global withdrawal syndrome score was separated out by time (such that for each behavior, adding up the values for each mouse across all time points equaled the normalized withdrawal symptom score for that behavior).

### UV CPP testing

Procedures were followed as described (*7*). Briefly, the apparatus used for CPP assays consisted of a chamber with black interior and dim lighting and a chamber with white interior and bright lighting, connected by a smaller gray “neutral” chamber. Before baseline preference assessments, mice were pretreated with UVB as indicated above. Baseline place preferences before conditioning were assessed by placing the mice in the neutral chamber and recording the times spent in the white chamber over the next 10 min. On the subsequent 8 days, conditioning took place in which mice were exposed to either UVB (50 mJ/cm^2^) or mock UV 15 min before placing them in the white chamber, and all animals were exposed to mock UV 15 min before placement in the black chamber. Conditioning time in each chamber was 30 min. For each mouse, there were at least 4 hours between the two conditioning sets. A day after the last conditioning, place preferences were tested again for 10 min.

### Morphine CPP testing

For morphine CPP, similarly to UV-CPP, baseline place preferences were assessed before conditioning. Then, conditioning took place in which mice were conditioned with either different doses of morphine (5, 10, or 20 mg/kg) or saline injected intraperitoneally in the white chamber, and all animals were conditioned with saline in the black chamber. Conditioning time in each chamber was 30 min right after injections. A day after the last conditioning, place preferences were tested again for 10 min.

For the alternative CPP setup, we used an unbiased approach (*35*). For these experiments, a different apparatus was used that has different tactile cues (metal rods and a mesh) in the black and white compartments, allowing for equal preferences for both sides of the apparatus. For testing baseline preferences on day 1, the mice were placed in the middle gray chamber and habituated for 5 min. Afterward, the doors to both side chambers were lifted, and the times spent in the black and white chambers were recorded for a total of 20 min.

For conditioning sessions, mice were paired to a chamber based on their baseline preferences in an unbiased manner in respect to the side of the chamber and to the initial preference. On days 2 and 4, all mice were intraperitoneally injected with PBS and were then conditioned in the “unpaired” chamber for 30 min. On days 3 and 5, the mice were injected either PBS or morphine (0.25 mg/kg) and were conditioned in the “paired” chamber for 30 min. Forty-eight hours after, the last injection preferences were tested again, similarly to day 1.

For CPP assay using calcitriol, calcitriol (10 μg/kg) or vehicle (20% ethanol, 30% propylene glycol, and 50% H_2_O) was intraperitoneally injected in 100 μl daily for 3 days prior starting the CPP assay until the last conditioning session (eight injections total). CPP was performed as in Fig. 1, using morphine (20 kg/mg).

### Nicotine CPP testing

Nicotine CPP was conducted similarly to UV CPP. Baseline preferences were assessed for 15 and 20 min for the experiments using young (average 8 weeks old) and old (average 8 months old) mice, respectively. Nicotine bitartrate salt (pH adjusted for 7.2) was used in a 0.25 mg/kg dose (dose was calculated for the nicotine base form). Mice were conditioned with either nicotine or saline injected subcutaneously in the white chamber, and all animals were conditioned with saline in the black chamber. Conditioning time in each chamber was 30 min right after injections. Only one conditioning was performed per day. A day after a total 4 days of conditionings (two with saline, two with saline/nicotine), place preferences were tested again.

### Quantitative polymerase chain reaction and western blot

To obtain nucleus accumbens and VTA samples, mice were intraperitoneally injected with morphine (5 mg/kg) or saline and were cervically dislocated at different time points (30, 60, 90, or 180 min) after injections. Mice receiving saline injections were euthanized 60 min after injection and were used as control groups for all morphine-treated groups. Brains were then immediately flash frozen in isopentane and stored in −80°C.

Punches were taken from coronal sections of the mouse brain for both NACC and VTA. For NACC, the *aci* and *fmi* white matter structures were used as landmarks to determine Bregma 1.94. Bilateral punches with a diameter of 1.5 mm were then taken 2 mm in depth with *aci* at the center of the punch, to ensure that both the accumbens core and shell were within the punch. To reach the VTA, coronal sections were taken until white matter structures of the cingulum (*cg*) were rounded and the dentate gyrus of the hippocampus began to form ventrally and caudally, as observed at Bregma of −2.92 mm. Bilateral punches with a diameter of 1.5 mm were then taken at ~1.0 mm in depth at a location of 0.5 mm lateral and from the midsagittal plane and 0.5 mm from the most ventral portion of the brain. Location of punches is displayed in fig. S13. RNA and proteins were then isolated as recently published (*36*).

### RNA sequencing

The extracted RNA samples were checked for overall quality, and only samples with high quality [RNA integrity number (RIN) ≥ 8.0] and high purity (optical density of 260/280 = 1.8 to 2.0) were used to generate libraries using the Bioo Scientific NEXTflex Rapid Directional RNA-seq Library Prep Kit (Bioo Scientific, Auston, TX). The final library was quantitated using the Agilent 2200 TapeStation High Sensitivity DNA Analysis (Agilent Technologies, Santa Clara, CA) for size and Qubit dsDNA HS Assay (Life Technologies, Waltham, MA) for concentration. The resulting mRNA libraries were then sequenced at the John Wayne Cancer Institute Sequencing Center on an Illumina HiSeq 2500 in Rapid Mode using 101-bp paired-end reads.

### RNA-seq analysis

Raw RNA-seq reads were first checked for overall quality using FastQC (www.bioinformatics.babraham.ac.uk/projects/fastqc/) and filtered for adapter contamination using Trimmomatic (version 0.36) (*37*) before downstream analysis. The filtered reads were then mapped to the GENCODE mouse comprehensive gene annotation set (version CGRm38) using the STAR aligner (version 2.4.2a) (*38*) with default parameters. Gene level read counts were generated using the *quantMode GeneCounts* option in STAR. Significantly differentially expressed genes were identified using the Bioconductor package DESeq2 (*39*) with a significance threshold of false discovery rate < 0.05. The upstream regulator analyses and pathway analyses were generated through the use of Ingenuity Pathway Analysis (QIAGEN Inc., www.qiagenbioinformatics.com/products/ingenuity-pathway-analysis (*40*). Gene Set Enrichment Analysis (GSEA) was performed using standard setting in GSEAv2.2.

### VDR expression analysis from human brain samples

Six human postmortem brain transcriptomic profiles were downloaded from the Allen Human Brain Atlas (*41*) in May 2017. mRNA enrichment analysis was done as described previously (*42*). Only those 190 brain regions were used in the analysis where data were available from at least four donors. VDR expression was averaged per brain region per patient for *z* transformation. Generated *z* values for individual brain regions represent the difference in the number of SDs compared with the average expression across all brain regions. Brain regions were defined as VDR high (*z* > 1) and VDR low (*z* < 1).

### c-fos staining of mouse brain samples

Mice were perfused with ice-cold PBS followed by 4% paraformaldehyde. Brains were fixed in 4% paraformaldehyde at 4°C for 48 hours. Brains were sliced at 50-μm thickness using a Leica vibratome (VT 1000s). Slices were washed three times in PBS for 5 min, blocking with 6% goat serum + 2% bovine serum albumin + 0.3% Triton X-100 (Blocking solution) at room temperature for 1 hour. Slices were then incubated with primary antibody (1:500; rabbit anti–c-Fos, Cell Signaling) in blocking solution at 4°C for overnight. After washing slices with PBS for three times, slices were incubated with second antibody (1:2000; goat anti-rabbit Alexa 488, Jackson ImmunoResearch). Images displayed in fig. S11 were acquired with a Nikon A1 confocal microscope.

For fluorescence quantification, ×4 magnification was used, and images were processed initially using NIS-Elements AR 5.02.00 64 bit and subsequently in ImageJ 1.46r. We selected only four regions (displayed in fig. S11A) for quantification that showed the most potential difference in c-fos staining out of nine regions investigated [ACC, NAC core, striatum, hippocampus, BLA, periaqueductal grey area (PAG), VTA, mammilary body (MM), and prefrontal cortex were considered]. Quantification was performed in ImageJ on 4× images after subtracting out background autofluorescence.

### Epidemiology

The NHANES is an annual population survey of the civilian U.S. population that contains both survey and physical examination data from more than 18,000 subjects, including information about current opioid painkiller use and serum VitD levels (NHANES 2003–2004). It uses a stratified multistage probability sampling design. Clinical information and laboratory data of the NHANES were available from the publicly accessible U.S. CDC database. The NHANES was approved by the Institutional Review Board (IRB), and documented consent was obtained from participants.

In our analysis of interest, randomly selected individuals aged 20 to 85 years were interviewed to obtain demographic characteristics, relevant medical conditions, and other clinical information. Data on previous history of fracture, chronic pain for more than 1 year duration, opioid analgesic consumption, as well as VitD levels in the cycle year 2003–2004, were retrieved and analyzed. VitD levels were assayed using an equilibrium radioimmunoassay procedure by a central laboratory with quality assurance and monitoring.

Opioid analgesic consumption was identified by physical screening of prescribed medications or prescription scripts by interviewers. This was done among participants who replied that they had taken a medication in the past month for which they needed a prescription. The medication containers of all the products used or scripts were physically screened for consumption of opioid painkillers.

Analysis of the relationship between opioid analgesic consumption and VitD levels was done using χ^2^ or Fisher’s exact tests. Multivariate analysis with logistic regression modeling was performed with opioid analgesic consumption as the dependent variable and with age, gender, ethnic groups, fracture history, chronic pain (≥1 year), season of blood drawn (1 November to 30 April versus 1 May to 31 October), and VitD levels as the independent variables. Although opioid use has been shown to vary with geographic residence (*43*, *44*), we did not consider it as an independent variable, as serum VitD levels are associated with geographical residence (*45*, *46*). The levels were classified into deficient (<12 ng/ml), insufficient (12 to <20 ng/ml), and normal (≥20 ng/ml) levels, based on Institute of Medicine guidelines (*47*).

The regression models were built using a backward variable selection method with a threshold of α > 0.15 to remove any nonsignificant variables. VitD levels were included in all of our models, as it is the variable of interest. The statistical package for social sciences (IBM SPSS version 22) was used to evaluate statistical significance. ORs, 95% CI, and *P* values were calculated to test the null hypothesis of no association between VitD levels and opioid analgesic consumption. A two-sided *P* < 0.05 was considered statistically significant.

### MGH-based study

We assessed whether cases and controls were equally likely to have VitD measurements and had similar numbers of measurements available using χ^2^ tests. We assessed differences by case status using each patient’s most recent measurement and all available measurements in separate analyses. We compared VitD measurements between cases and controls as a continuous variable using *t* tests. We categorized VitD levels as optimal (>30 ng/ml), sufficient (21 to 30 ng/ml), insufficient (12 to 20 ng/ml), and deficient (<12 ng/ml). We compared the distribution of VitD levels between cases and controls using χ^2^ tests. Linear regression was used to assess the effect of case status on VitD measurement controlling for demographics.

Cases and controls were equally likely to have VitD measurements (*P* = 0.951). Individuals with no measurements were not considered further. The remaining patients did not differ by case status on gender, race, age, or the number of available measurements. The number of VitD measurements were similar in patients with OUD compared to controls (*P* = 0.342). The study was approved by the IRB.

### Statistics

Prism 6.0 was used for statistical analysis. IBM SPSS version 22 was used for the epidemiology analysis.

## SUPPLEMENTARY MATERIALS

Supplementary material for this article is available at http://advances.sciencemag.org/cgi/content/full/7/24/eabe4577/DC1

## Appendix

View/request a protocol for this paper from *Bio-protocol*.
